# Supplementary material for: Metal3D: a general deep learning framework for accurate metal ion location prediction in proteins
Source: Nat Commun. 2023 May 11;14:2713. doi: 10.1038/s41467-023-37870-6 (PMC10175565; doi:10.1038/s41467-023-37870-6)
Supplement: Supplementary file 1 — Supplementary information [file 41467_2023_37870_MOESM1_ESM.pdf]

Metal3D: A general deep learning framework for accurate metal ion location prediction in proteins

Simon L. Dürr[1], **Andrea Levy**[1], **Ursula Rothlisberger**[1]

***For correspondence:**
ursula.roethlisberger@epfl.ch (UR)

[1]Laboratory of Computational Chemistry and Biochemistry, Institute of Chemical Sciences and Engineering, Swiss Federal Institute of Technology (EPFL) CH-1015 Lausanne, Switzerland

Github:
lcbc-epfl/metal-site-prediction
Webapp:
hf.space/simonduerr/metal3d
Interactive manuscript:
lcbc-epfl.github.io/metal-site-prediction

Supplementary information

Supplementary Table 1. Atoms used as reference points for each amino acid in Metal1D. In the case of amino acids with more than one possible ligand atom, the search radius is enlarged, the increase is computed from the midpoint between all ligating atoms. Typical values computed for structure data files downloaded from the PDB are reported.

Amino acid	Residue name	Label(s)	Search radius increase (Å)
Alanine	ALA	O	0
Arginine	ARG	NH1, NH2	1.2
Asparagine	ASN	OD1	0
Aspartic acid	ASP	OD1, OD2	1.105
Cysteine	CYS	SG	0
Glutamic acid	GLU	OE1, OE2	1.105
Glutamine	GLN	OE1	0
Glycine	GLY	O	0
Histidine	HIS	ND1, ND2	1.08
Isoleucine	ILE	O	0
Leucine	LEU	O	0
Lysine	LYS	NZ	0
Methionine	MET	SD	0
Phenylalanine	PHE	O	0
Proline	PRO	O	0
Serine	SER	OG	0
Threonine	THR	OG1	0
Tryptophan	TRP	O	0
Tyrosine	TYR	OH	0
Valine	VAL	OH	0

Supplementary Table 2. Atom selections used for voxelization of proteins using moleculekit

Channel name	Selected atoms
aromatic	HIS TRP TYR PHE sidechain without CB
hydrophobic	element C

Channel name	Selected atoms
occupancy	all protein heavy atoms
hbond donor	(ASN GLN TRP MSE SER THR MET CYS and name ND2 NE2 NE1 SG SE OG OG1) and name N
hbond acceptor	(resname ASP GLU HIS SER THR MSE CYS MET and name ND2 NE2 OE1 OE2 OD1 OD2 OG OG1 SE SG) or name O
metalbinding	(name ND1 NE2 SG OE1 OE2 OD2) or (protein and name O N)
positive charge	resname LYS ARG HIS and name NZ NH1 NH2 ND1 NE2 NE
negative charge	resname ASP GLU and name OD1 OD2 OE1 OE2

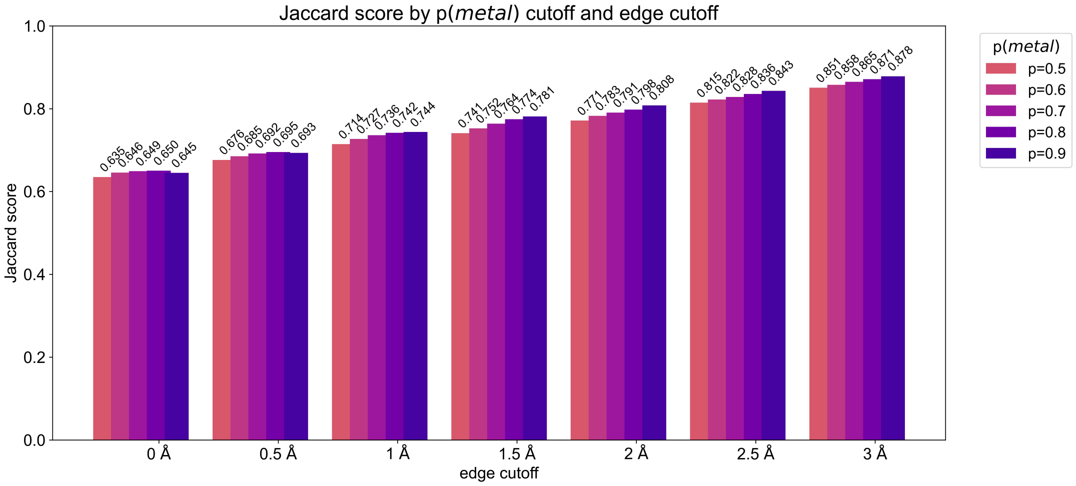

Supplementary Figure 1. Jaccard Index: Discretized Jaccard indices using different cutoffs for edge trimming and different probability cutoffs (p(metal)) showing that Metal3D predictions well reproduce the target environments in the test set.

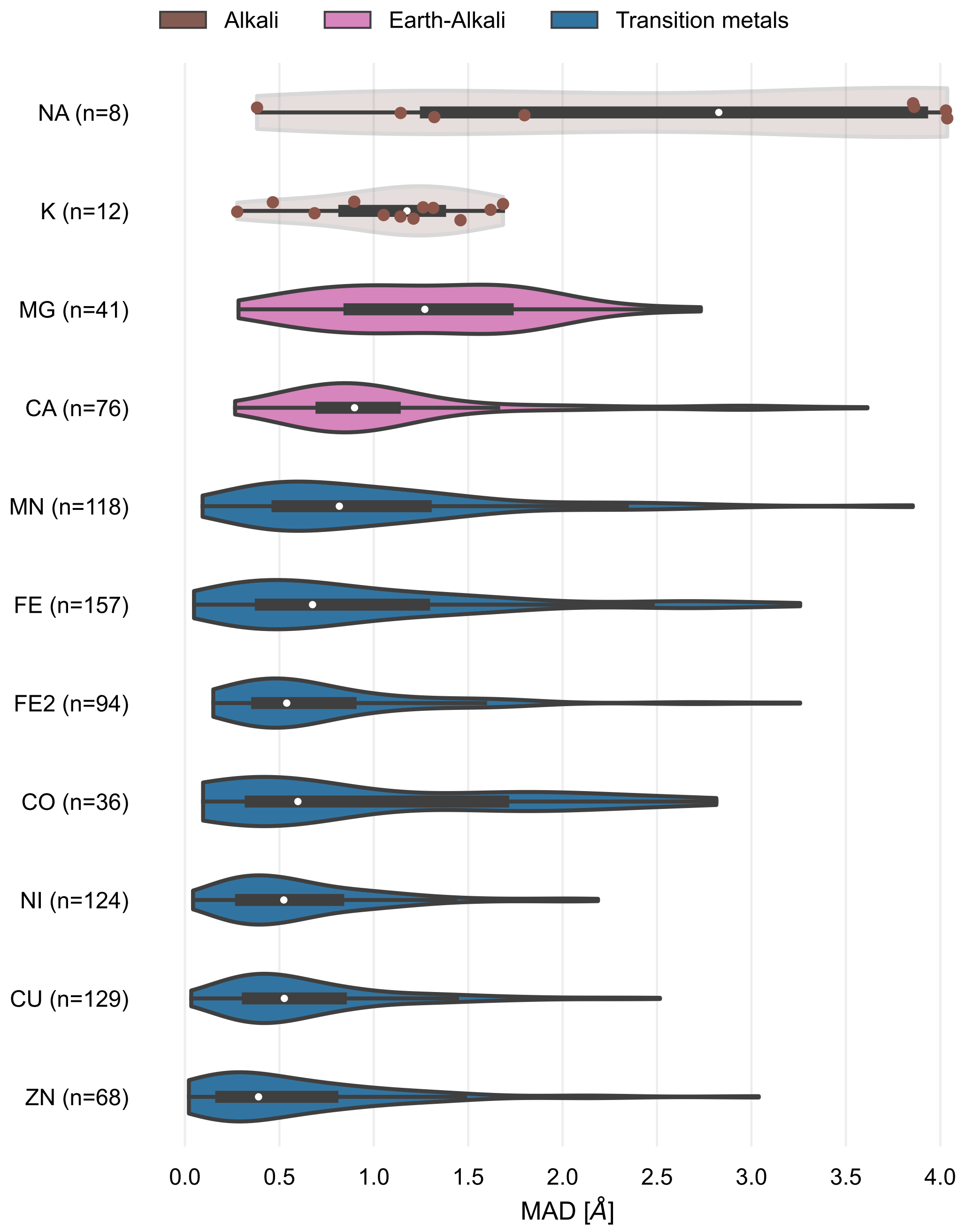

Supplementary Figure 2. MAD for Metal3D for all sites with 3+ unique protein ligands and occupancy > 0.5 in the test set and for the selected structures for the other metals. For each ion the box plot indicates the median distance deviation (white dot) and the first quartiles (black box). The kernel density estimation of all data points is shown as violin plot with minima and maxima indicated by whiskers. For alkali ions due to low sample size all individual points are also shown.

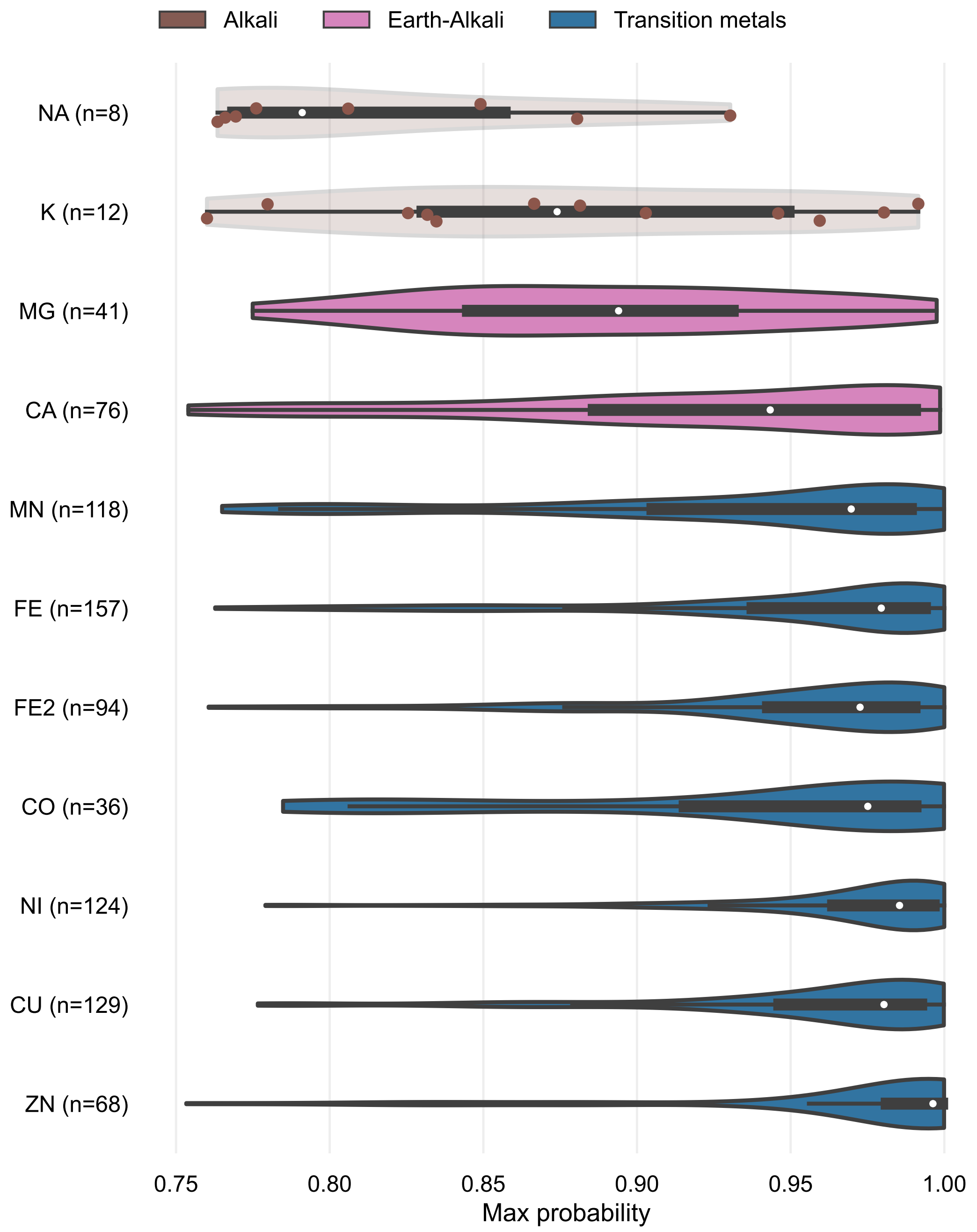

Supplementary Figure 3. Probability distribution for Metal3D on sites with 3+ unique protein ligands and occupancy > 0.5 in the test set and for the selected structures for the other metals. For each ion the box plot indicates the median probability (white dot) and the first quartiles (black box). The kernel density estimation of all data points is shown as violin plot with minima and maxima indicated by whiskers. For alkali ions due to low sample size all individual points are also shown.

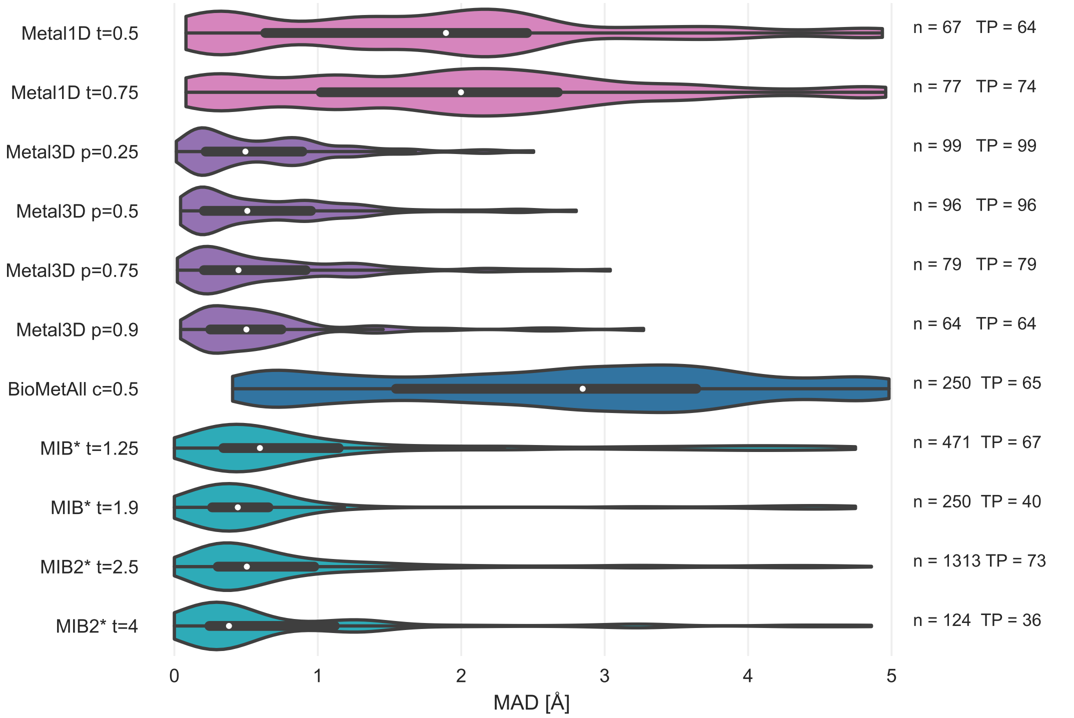

Supplementary Figure 4. MAD only 2+ residue coordinated zincs with occupancy > 0.5 in the test set. For each tool the box plot indicates the median distance deviation (white dot) and the first quartiles (black box). The kernel density estimation of all data points is shown as violin plot with minima and maxima indicated by whiskers. n indicates the number of individual locations predicted within 5 Å of the zinc, TP the number of found sites

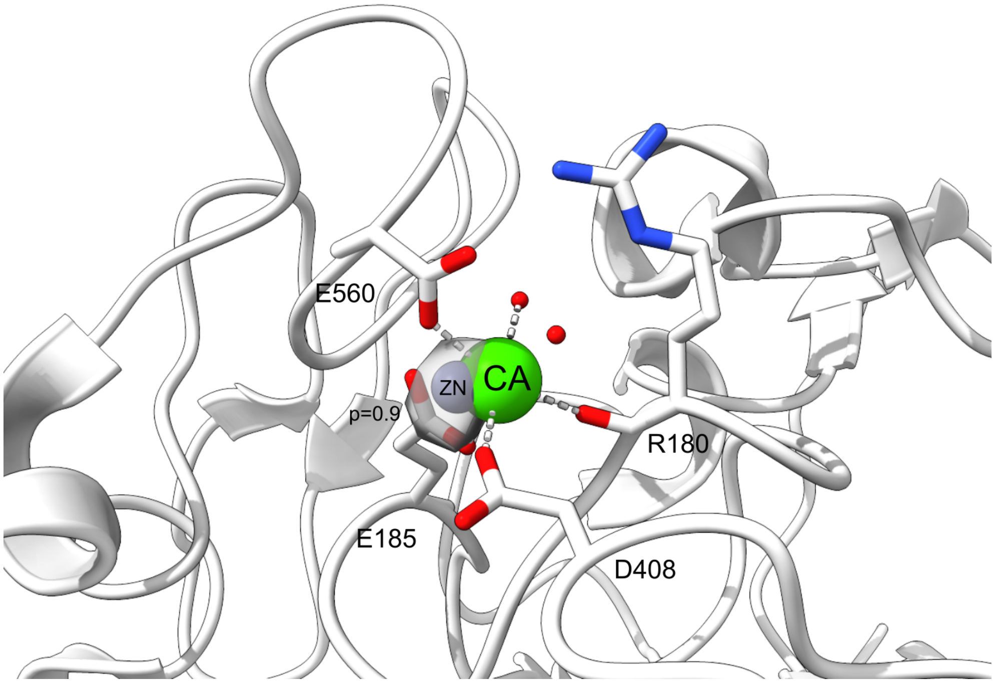

Supplementary Figure 5. False positive for Metal3D at p=0.9 in PDB 4JJJ: A calcium site is misclassified as zinc site.

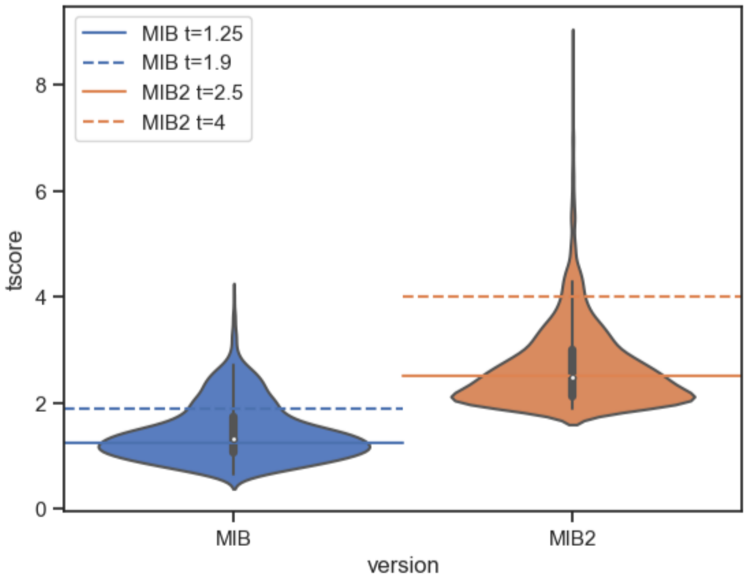

Supplementary Figure 6. MIB2 & MIB t-score distribution: MIB2 is an updated version of MIB with extended template database. Due to more templates in the database the t-score distribution of the predictions on the testset is different. The old and new t-scores are highlighted with straight and dashed line. The kernel density estimation of all t-scores is shown as violin plot with minima and maxima indicated by whiskers, the boxplot indicates the median t-score as white dot and first quartiles are shown in the black box. MIB n=1188, MIB2 n=3350

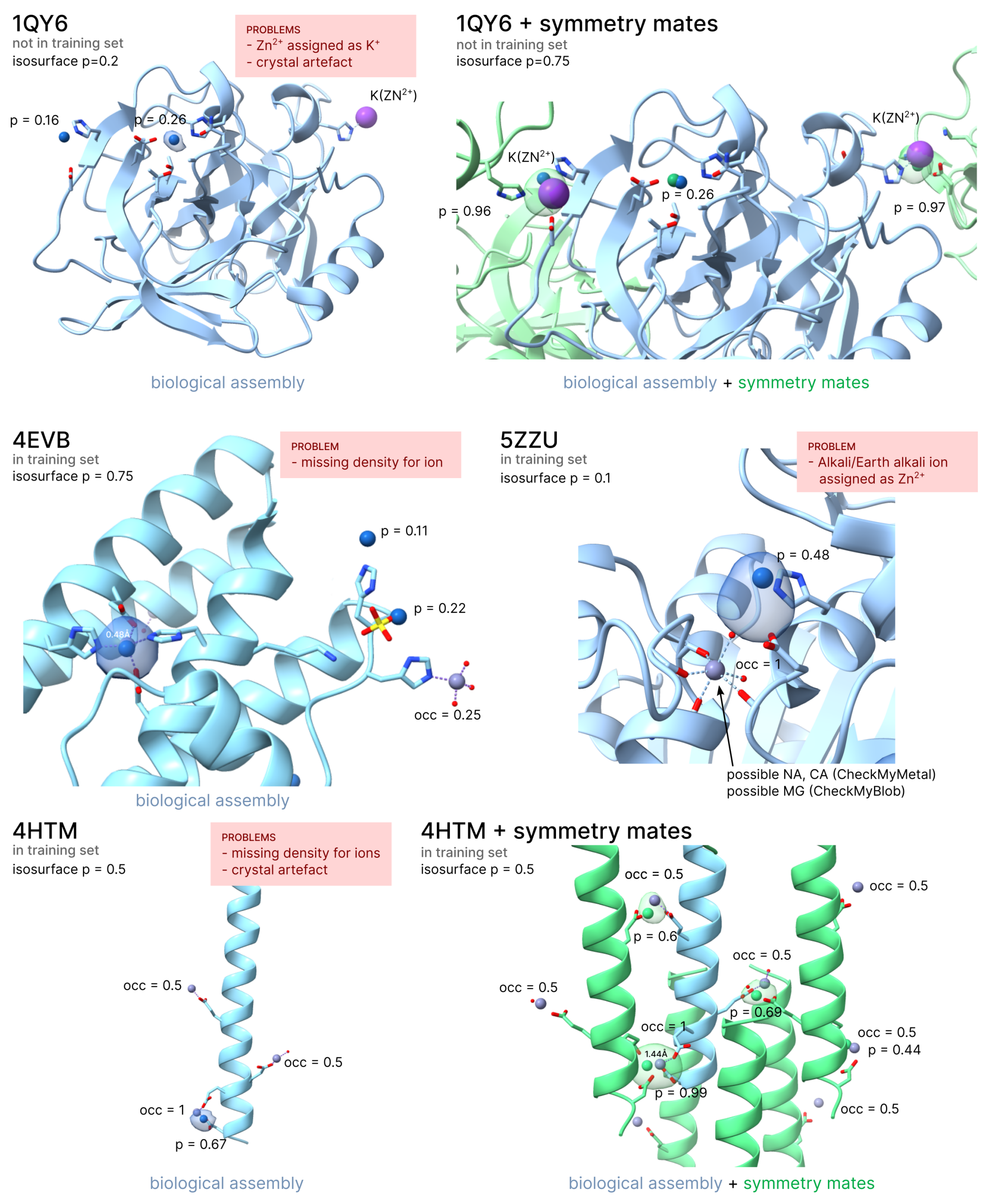

Supplementary Figure 7. Performance of Metal3D on wrong metal sites. 1QY6: Deposited structure contains a Zn^{2+} site at a crystal contact wrongly assigned as K^{+}. Metal3D only identifies the site as zinc site with high probability if the symmetry mates are present. **4EVB:** Metal3D was explicitly trained on all the Zn^{2+} sites in this structure. In inference, the site with occupancy 0.25 is not predicted showing that Metal3D ignores issues with data quality in the training set. **5ZZU:** An alkali/earth-alkali binding site (e.g. NA, CA or MG according to CheckMyMetal[1], CheckMyBlob [2]) wrongly assigned as Zn^{2+}. In inference, Metal3D does not predict this site even though it was trained on this site. **4HTM:** All metal ion binding sites in this structure are crystal contacts. Metal3D was trained on this structure and only predicts the metal ion binding site that has 2 protein ligands using the biological assembly. When symmetry mates are present the other sites with coordination partners from symmetry adjacent structures are also predicted. Biological assemblies in blue, symmetry mates in green, zinc sites as purple, predicted locations blue. Occupancy (occ=) and maximum predicted probability (p=) indicated for select sites.

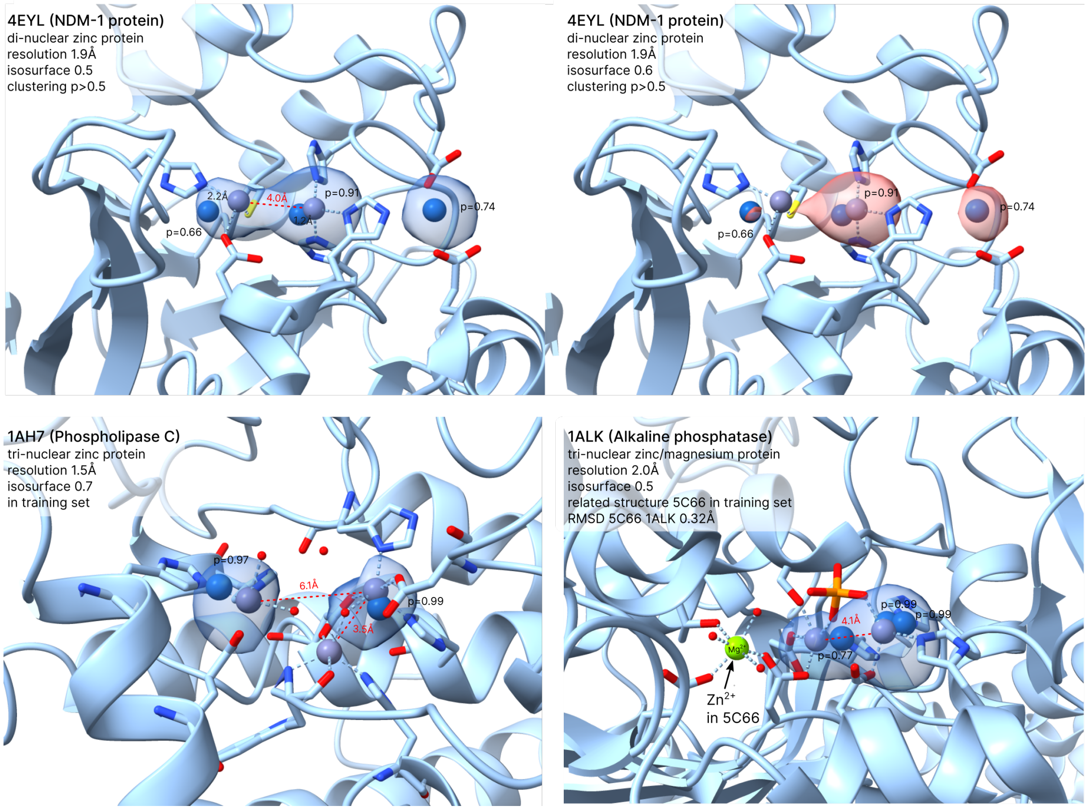

Supplementary Figure 8. Multi-nuclear metal sites. 4EYL: Metal3D probability density has density for the two modelled zinc ions and one FP. The probability density separates into two separate density blobs with p>0.5 (left panel). For placing separate ions using clustering higher probability cutoff p>0.5 is required. **1AH7:** Two of 3 experimental zincs have probability density predicted by Metal3D. One ion with backbone coordination is not found. **1ALK:** The two zinc ion binding sites are correctly found by Metal3D with correctly placed probes at default cutoff. The Mg^{2+} site which can also bind Zn^{2+} (as is the case for 5C66 in the training set) is not predicted even though the model was trained on it. Predicted ion location placed by Metal3D after clustering voxels in dark blue. Experimental Zn^{2+} in purple.

Dataset statistics

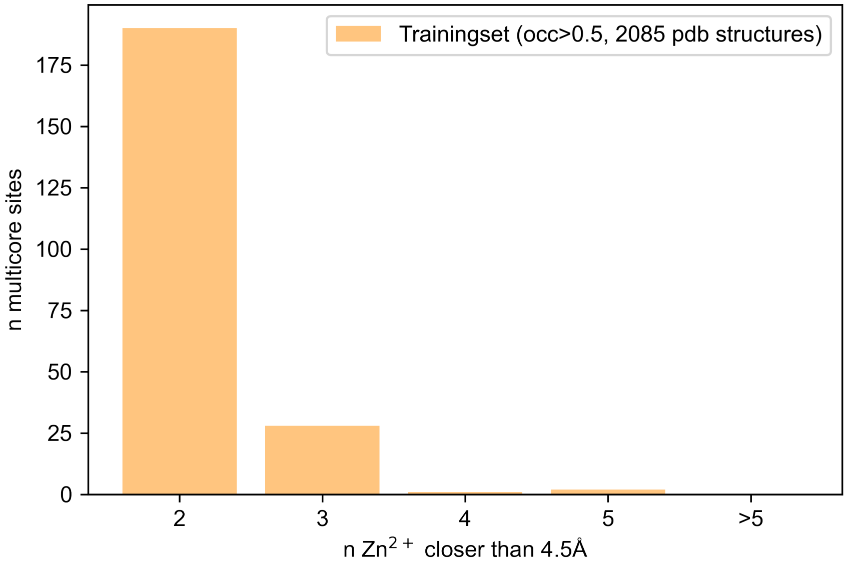

Supplementary Figure 9. Number of multicore Zn^{2+} sites in the biological assemblies included in the training set. Two sites are assigned to the same multicore site if their distance is lower than 4.5 Å. Sites with occupancy lower than 0.5 have been excluded.

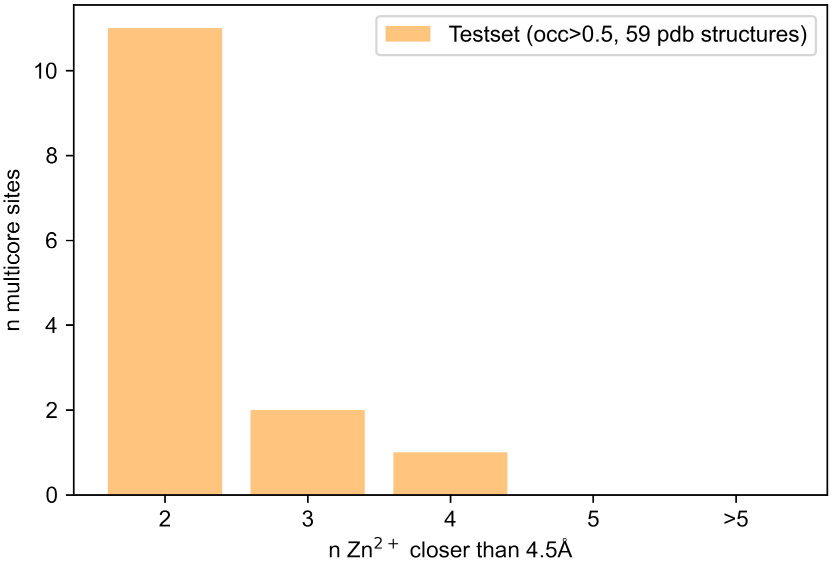

Supplementary Figure 10. Number of multicore Zn^{2+} sites in the biological assemblies included in the test set. Two sites are assigned to the same multicore site if their distance is lower than 4.5 Å. Sites with occupancy lower than 0.5 have been excluded.

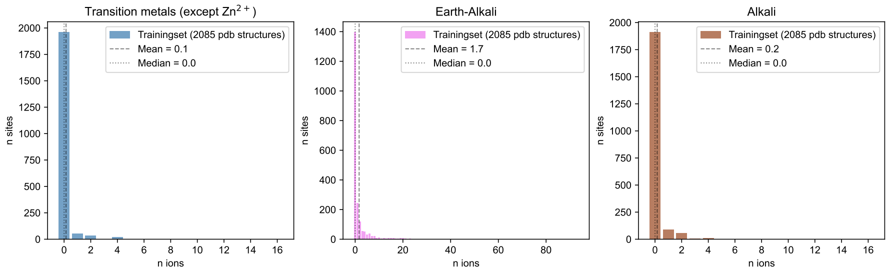

Supplementary Figure 11. Number of metal ions for the biological assemblies included in the training set. The same types of transition (Fe^{2+}, Fe^{3+}, Co^{2+}, Cu^{2+}, Cu^{+}, Mn^{2+}), earth-alkali (Mg^{2+}, Ca^{2+}), and alkali (Na^{+}, K^{+}) metals used in the Selectivity for other metals section is used. For each plot, the mean and the median values are highlighted by a dashed and dotted line, respectively.

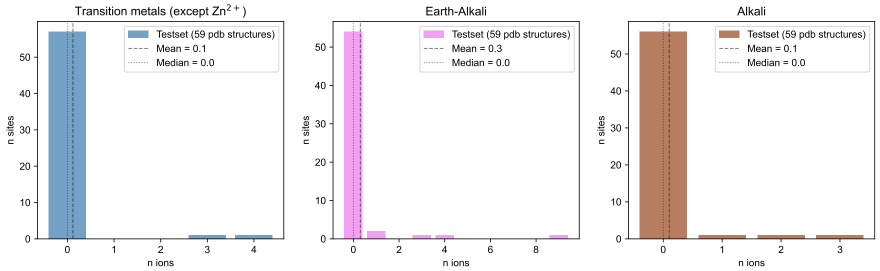

Supplementary Figure 12. Number of metal ions for the biological included in the test set. The same types of transition (Fe^{2+}, Fe^{3+}, Co^{2+}, Ni^{2+}, Cu^{2+}, Mn^{2+}), earth-alkali (Mg^{2+}, Ca^{2+}), and alkali (Na^{+}, K^{+}) metals used in the Selectivity for other metals section is used. For each plot, the mean and the median values are highlighted by a dashed and dotted line, respectively.

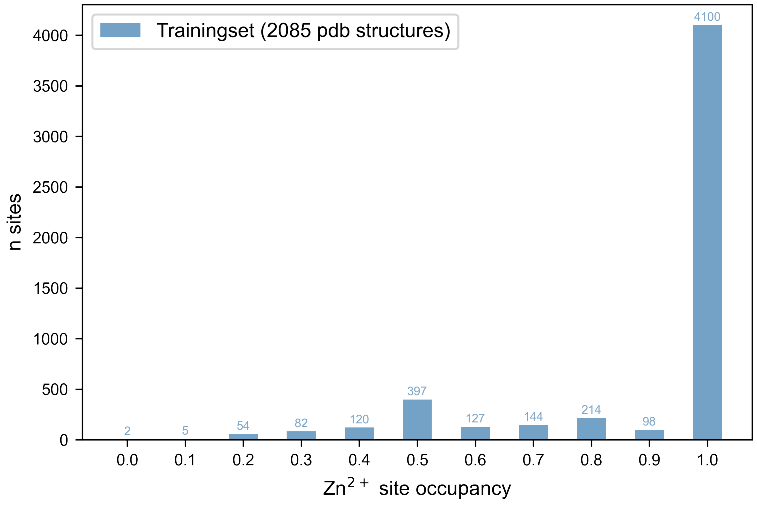

Supplementary Figure 13. Occupancy values for Zn^{2+} ions contained in the biological assemblies included in the training set. The same quantity is also reported for filtered versions of the training set, i.e. 2+ coord. ZN (sites with occupancy larger than 0.5 binding at least two amino acids) and 3+ coord. ZN (sites with occupancy larger than 0.5 binding at least three amino acids)

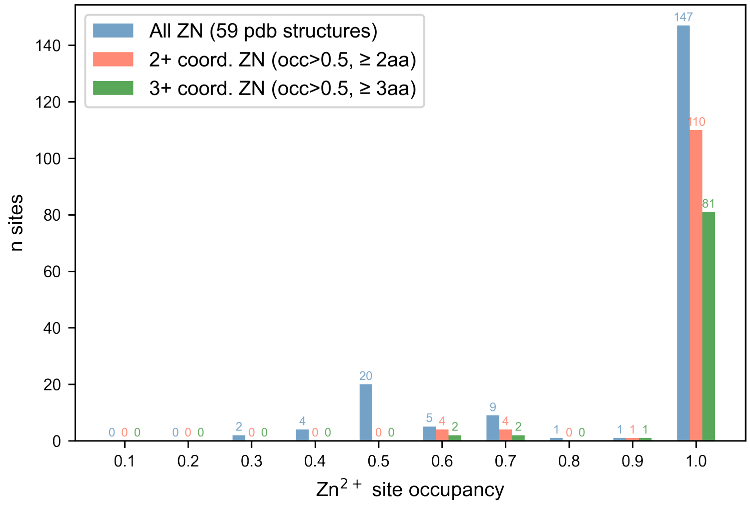

Supplementary Figure 14. Occupancy values for Zn^{2+} ions contained in the biological assemblies included in the test set. The same quantity is also reported for filtered versions of the test set, i.e. 2+ coord. ZN (sites with occupancy larger than 0.5 binding at least two amino acids) and 3+ coord. ZN (sites with occupancy larger than 0.5 binding at least three amino acids).

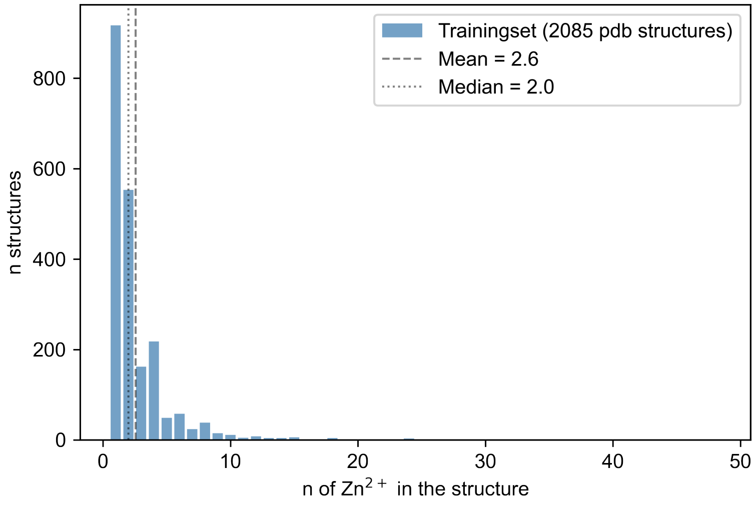

Supplementary Figure 15. Number of Zn^{2+} sites for the structures included in the training set. The mean (2.6) and median (2.0) value are highlighted by a dashed and dotted line, respectively.

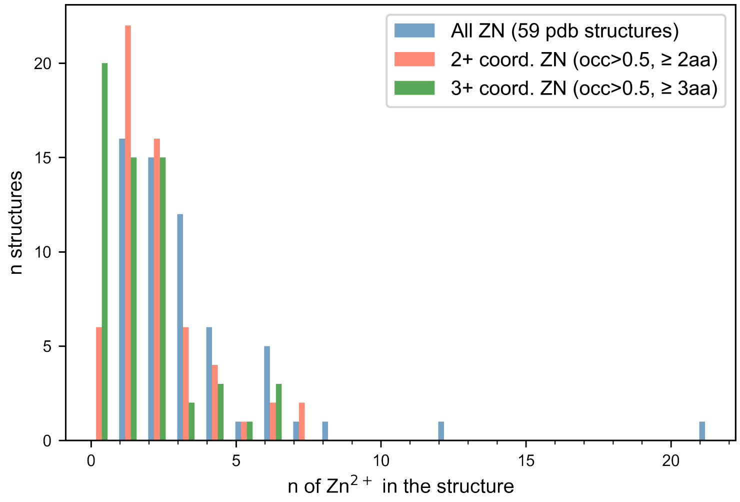

Supplementary Figure 16. Number of Zn^{2+} sites for the biological assemblies included in the test set. The same quantity is also reported for filtered versions of the test set, i.e. 2+ coord. ZN (sites with occupancy larger than 0.5 binding at least two amino acids) and 3+ coord. ZN (sites with occupancy larger than 0.5 binding at least three amino acids).

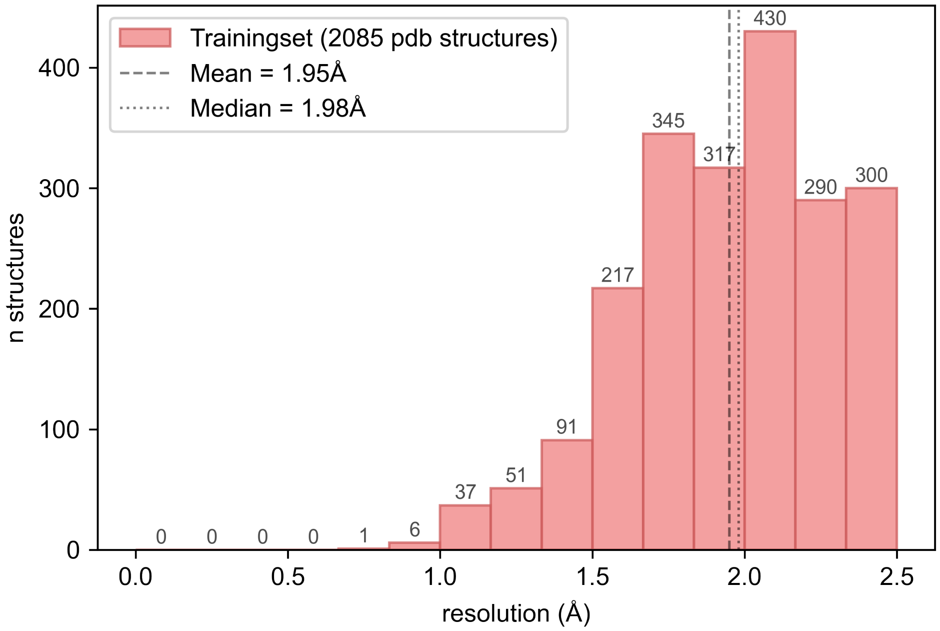

Supplementary Figure 17. Distribution of the resolution for the biological assemblies included in the training set. The mean (1.95 Å) and median (1.98 Å) value are highlighted by a dashed and dotted line, respectively.

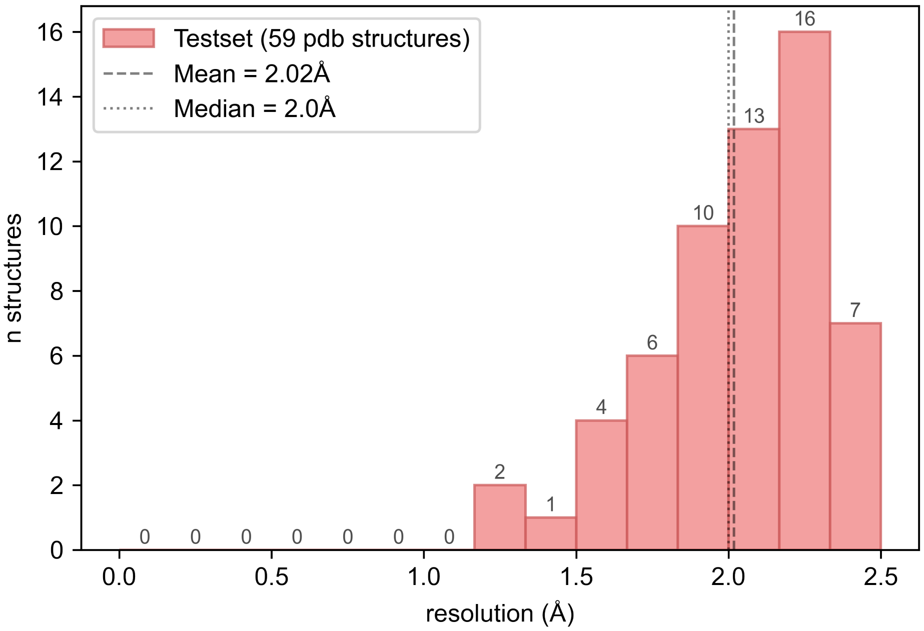

Supplementary Figure 18. Distribution of the resolution for the biological assemblies included in the test set. The mean (2.02 Å) and median (2.0 Å) value are highlighted by a dashed and dotted line, respectively.

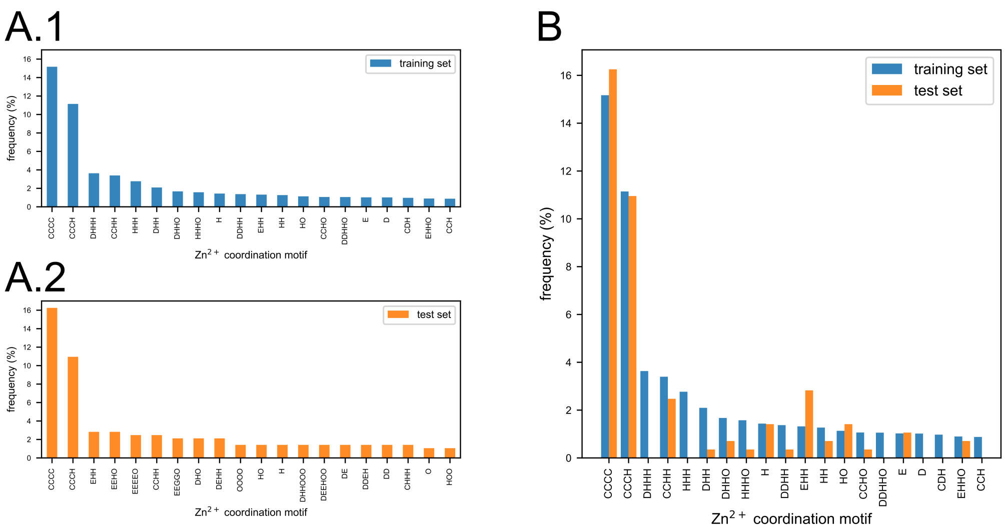

Supplementary Figure 19. Distribution of the coordination motifs. Biological assemblies included in the training set (A.1) and the test set(A.2) sorted by frequency and overlayed sorted by frequency of the coordination motifs in the training set (B).

Supplementary Table 3. MAD and median on zinc test set for all zinc sites (n=189).

tool	MAD [Å]	median [Å]
BioMetAll c=0.5	2.72 ± 1.33	2.86
MIB t=1.25	1.13 ± 1.24	0.60
MIB t=1.9	0.77 ± 1.09	0.44
MIB2 t=2.5	0.89 ± 1.00	0.52
MIB2 t=4	0.85 ± 1.02	**0.43**
Metal1D t=0.5	2.07 ± 1.33	2.06
Metal1D t=0.75	2.12 ± 1.26	2.12
Metal3D p=0.25	0.74 ± 0.66	0.61
Metal3D p=0.5	0.73 ± 0.66	0.54
Metal3D p=0.75	0.71 ± 0.64	0.51
Metal3D p=0.9	**0.70 ± 0.64**	0.52

Supplementary Table 4. MAD and median on zinc test set for zinc sites with at least 2 protein ligands and occupancy > 0.5 (n=119).

tool	MAD [Å]	median [Å]
BioMetAll c=0.5	2.67 ± 1.33	2.85
MIB t=1.25	1.08 ± 1.21	0.60
MIB t=1.9	0.77 ± 1.09	0.44
MIB2 t=2.5	0.87 ± 1.00	0.50
MIB2 t=4	0.79 ± 1.02	**0.38**
Metal1D t=0.5	1.82 ± 1.28	1.89
Metal1D t=0.75	1.95 ± 1.26	2.00
Metal3D p=0.25	0.64 ± 0.53	0.50
Metal3D p=0.5	0.67 ± 0.59	0.51
Metal3D p=0.75	0.68 ± 0.64	0.45

tool	MAD [Å]	median [Å]
Metal3D p=0.9	**0.66 ± 0.62**	0.50

Supplementary Table 5. Main parameter for each method. Arrows indicate in which direction higher quality results are obtained (lower recall but higher precision).

Method	parameter	description
Metal3D	Probability p ↑	Probability value predicted by the Metal3D model
Metal1D	Threshold t ↓	Threshold that is used twice for the scoring to remove residues in step 1 (residue scoring) that have score lower than $(1-t) \times$ max residue score and in step 2 (putative site scoring) to remove sites that have score lower than $(1-t) \times$ max site score
BioMetAll	Cluster cutoff c ↑	Denotes the number of probes predicted for a given cluster divided by the number of probes for the highest scoring cluster
MIB/MIB2	Template similarity t ↑	Measure of sequence and structure conservation of the template site in reference to the predicted site

Supplementary References

(1) Zheng H., Chordia M. D., Cooper D. R., Chruszcz M., Müller P., Sheldrick G. M., Minor W., Validation of metal-binding sites in macromolecular structures with the CheckMyMetal web server. *Nature Protocols* **2014**, *9* (1), 156-70.

(2) Kowiel M., Brezezinski D., Porebski P. J., Shabalin I. G., Jaskolski M., Minor W., Automatic recognition of ligands in electron density by machine learning *Bioinformatics* **2019**, *35*(3) 452-461 https://doi.org/10.1093/bioinformatics/bty626.
